# SBMLmod: a Python-based web application and web service for efficient data integration and model simulation

**DOI:** 10.1186/s12859-017-1722-9

**Published:** 2017-06-24

**Authors:** Sascha Schäuble, Anne-Kristin Stavrum, Mathias Bockwoldt, Pål Puntervoll, Ines Heiland

**Affiliations:** 1 Jena University Language & Information Engineering (JULIE) Lab, Friedrich-Schiller-University Jena, Jena, Germany; 2 Department of Informatics, University of Bergen, Bergen, Norway; 3 Department of Arctic and Marine Biology, UiT The Arctic University of Norway, Tromsø, Norway; 4 Centre for Applied Biotechnology, Uni Research Environment, Bergen, Norway

**Keywords:** Web application, Web service, Data integration, Model simulation

## Abstract

**Background:**

Systems Biology Markup Language (SBML) is the standard model representation and description language in systems biology. Enriching and analysing systems biology models by integrating the multitude of available data, increases the predictive power of these models. This may be a daunting task, which commonly requires bioinformatic competence and scripting.

**Results:**

We present SBMLmod, a Python-based web application and service, that automates integration of high throughput data into SBML models. Subsequent steady state analysis is readily accessible via the web service COPASIWS. We illustrate the utility of SBMLmod by integrating gene expression data from different healthy tissues as well as from a cancer dataset into a previously published model of mammalian tryptophan metabolism.

**Conclusion:**

SBMLmod is a user-friendly platform for model modification and simulation. The web application is available at http://sbmlmod.uit.no, whereas the WSDL definition file for the web service is accessible via http://sbmlmod.uit.no/SBMLmod.wsdl. Furthermore, the entire package can be downloaded from https://github.com/MolecularBioinformatics/sbml-mod-ws. We envision that SBMLmod will make automated model modification and simulation available to a broader research community.

**Electronic supplementary material:**

The online version of this article (doi:10.1186/s12859-017-1722-9) contains supplementary material, which is available to authorized users.

## Background

Theoretical models of complex biological entities are fundamental to systems biology and systems medicine research [1, 2]. They provide summaries of metabolic, signalling or gene regulatory networks including information on e. g. stoichiometry or kinetic rate laws. To gain new biological insights into pathways of interest it is nevertheless crucial to integrate experimental data. The type of appropriate data is context dependent: While dynamic signalling or metabolic pathway studies may require metabolome or time course data, gene regulatory networks commonly ask for gene expression datasets. Such data are increasingly available from data repositories such as the Gene Expression Omnibus (GEO) [3], the NCI-60 tumour cell line screens [4, 5] and The Cancer Genome Atlas (TCGA, https://cancergenome.nih.gov).

Theoretical model generation and distribution itself is commonly achieved via multiple toolboxes and databases. Pathway Tools [6] and CellDesigner [7] are examples of software packages for biological model construction. Whereas COPASI [8] and Data2Dynamics [9] are toolboxes for investigating dynamic behaviour, the COBRA toolbox [10] is suited for constraint-based model analyses. Theoretical models are stored in public databases such as the BioModels database [11], which mainly covers small to medium scale models, or the BiGG model database (http://bigg.ucsd.edu/) for genome-scale models. Model accessibility is achieved by model definition standards, such as the Systems Biology Markup Language (SBML) [12].

Both vast amounts of data and standardised models are readily available, yet integrating and analysing data with a given model can still be a discouraging task. Nevertheless, programmatic access is commonly necessary to perform more complex operations than loading and simulating the initial model.

In recent years software packages have been made available to simplify model manipulation and simulation tasks [10, 13–15]. A Taverna workflow published by Li et al. [14] focuses on reconstruction, model manipulation and simulation. Data integration is realised via accessing the enzyme kinetics database SABIO-RK [16], or via an in-house database for specific metabolomics and proteomics datasets. It does not, however, include the possibility to integrate gene expression data. Setting up the workflow itself requires programmatic configuration including resolving software dependencies on e. g. the libSBML package [17]. Yizhak et al. [13] introduced a method termed IOMA, which quantitatively integrates proteomic and metabolomic data with genome-scale metabolic models and calculates steady state solutions. IOMA assumes Michaelis-Menten-like kinetics and delivers steady state flux distributions, but no metabolite concentrations. GAM presented by Sergushichev et al. [15] provides a convenient network analysis platform to analyse metabolic networks. So far it covers four pre-assembled models and is specifically tailored towards identification of the most regulated subnetwork between two conditions.

These toolboxes are appropriate ways to create, modify or simulate theoretical models. Yet because they require a minimum level of programming proficiency, they are all effectively restrictive for scientists with little or no computational biology background.

We present and describe SBMLmod, a slim and easily accessible SBML model loading, data integrating and model simulation platform. SBMLmod can be accessed within any common web browser, circumventing the need to install or program software. Any valid SBML model and a dataset for parametrisation can be chosen to perform model modification and simulation operations. Advanced users can access SBMLmod programmatically via its Web Services Description Language (WSDL) interface. The WSDL interface circumvents the need to resolve software dependencies and allows for the integration of SBMLmod into analysis pipelines. Finally, the complete package can be downloaded, installed, set up locally and accessed from any Python shell prompt.

## Implementation

Every SBMLmod task is based on a theoretical biological model encoded in SBML, which might be downloaded from e. g. the BioModels database [11]. Single or multiple data sets on either kinetic rate law or species concentration can be provided by the user. Steady state simulations can be calculated by making use of the web service COPASIWS from COPASI [8] to obtain system wide concentration and flux solutions feasible at steady state. SBMLmod can be accessed as a web application or as a web service for customised workflows. The respective WSDL file guarantees the same functionality as the web application.

SBMLmod is written in Python 2.7. Accessing and modifying SBML models is enabled via libSBML [17]. All model modification and simulation features are computed on the fly and scale efficiently with the number of data sets and data volume.

### Web application guarantees OS independent access of SBMLmod

**Fig. 1:**
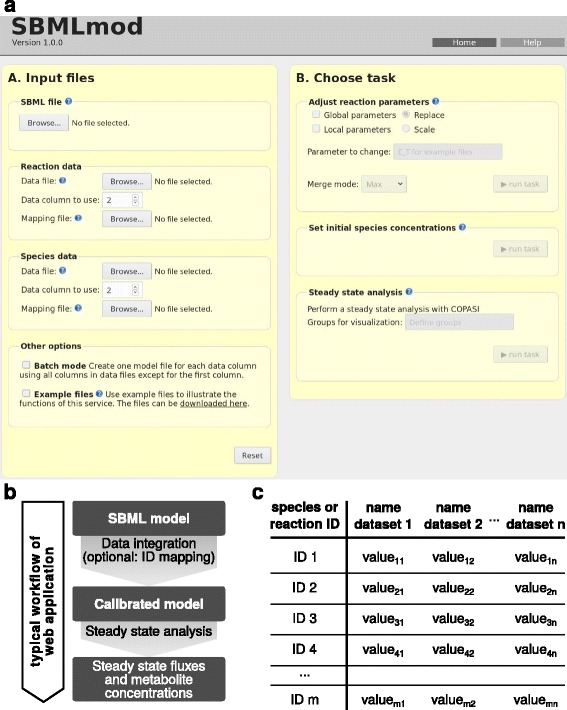
SBMLmod: basic workflow and input data outline. **a** Welcome screen of the web application. SBMLmod is organised into two panels. Input files are chosen in panel **a**. Mapping files are optional. Model modification and/or steady state analysis may be chosen in panel B. **b** Simplified workflow scheme of web application. An SBML model might be calibrated based on available data. Optionally, IDs might be mapped, if SBML model and data differ in the used identifier standard. Steady state concentration of metabolites and reaction flux analysis is feasible with COPASIWS [8]. **c** Basic outline of data file format. The first column comprises data specific IDs (e. g. gene identifier). The first row contains identifiers of the data in the respective column

The welcome screen of SBMLmod’s web application is organised into two panels: A) choosing the input files; B) choosing the task to perform (Fig. 1 a). The general workflow is shown in Fig. 1 b.

Input files are comprised of a mandatory SBML model file and optional data files. The latter may concern either parameters of reaction rate laws or the initial concentrations of considered species in the model. An additional mapping file is mandatory whenever the identifiers given in the data file do not match the identifiers of the respective species or reaction in the model file. This may be the case, if, for instance, different identifier standards (e. g. ensembl, or entrez gene id) are used in the model and data file(s), or if different synonyms for the same species or reaction are used.

Users may furthermore choose to analyse multiple data sets by selecting the ‘batch mode’ option. If selected, each column of a given data file is processed individually and will yield a separate data specific model or simulation.

After selecting the necessary files, the user can either calibrate or simulate the given model by selecting the respective options (Fig. 1 a, panel B). Calibrating the model parameters is accomplished by replacing or scaling reaction parameters such as the total amount of available enzyme concentrations. Replacing and scaling reaction parameters can be accomplished system-wide (globally) or on a per-reaction basis (locally). Should multiple rows of a given data file be associated with the same reaction (e. g. if isozymes are considered in the data file, but not in the model), the user may choose a specific merge mode. All merge options (e. g. maximum value selection) are described in detail in the online documentation and in the Additional file 1: S1. The initial concentrations of model species can also be modified. The most recently modified models are always available for download. They are identified by the respective column header in the data file (cf. Fig. 1 c and Additional file 1: S1 for details on the data file format).

A warning feedback functionality is established and ensures that models are correctly encoded, all identifiers are assignable and mappings are unambiguous. The web application of SBMLmod is set up using Python Django [18] and is hosted at http://sbmlmod.uit.no. To demonstrate data format and warning feedback, example files are available at the website and in Additional file 2: S2.

Calculation of steady state concentrations and fluxes are enabled by linking the web application to the COPASI web service. Our web application returns the original output file(s) generated. In addition, results of generated and simulated models (in batch mode) are returned as accumulated, tab separated tables for the calculated concentrations and fluxes. To allow an initial inspection of the results, the web application generates a customisable graph showing all non-constant metabolite concentrations and fluxes (cf. Additional file 3: Figure S3 for an example output). Customisation includes selecting metabolite species and fluxes to be shown and also allows for grouping together different values (if batch mode was selected). See Additional file 1: S1 for details of customisation options.

### Web service accessibility enables automated high throughput data integration and analysis

Next to the web application, a web service functionality of SBMLmod is available. It can be accessed via the WSDL interface, either from http://sbmlmod.uit.no/SBMLmod.wsdl or by downloading the whole package including the WSDL file at https://github.com/MolecularBioinformatics/sbml-mod-ws. The web service enables complete analysis workflows including a full sequence of model modification and simulation operations of the aforementioned features. By providing the WSDL file, we enable more advanced users to run data integration without the need to install software packages and resolve software dependencies. SBMLmod can thus be integrated into other existing or newly developed workflows for model manipulation or steady state simulation. Alternatively the web service can be installed and run locally (source files and technical documentation are available at https://github.com/MolecularBioinformatics/sbml-mod-ws). This enables faster processing especially for large datasets. Simulation results are summarised in textual output files. These can be further processed using our Python toolbox PyCopasi for parsing and manipulating COPASI files. PyCopasi is available at https://github.com/MolecularBioinformatics/PyCopasi.

Feasible model manipulations and basic scripts to run the data integration are exemplified by files provided in the ‘testClient’ folder of the package.

## Results & discussion

To demonstrate the usage of SBMLmod we analysed two publicly available datasets by integrating them into an existing model of tryptophan metabolism [19] (https://www.ebi.ac.uk/biomodels-main/MODEL1310160000). Tryptophan, an essential amino acid, has received increasing interest in recent years, since it is the precursor of several bioactive metabolites such as serotonin, kynurenine, melatonin and NAD. Consequently, imbalances in tryptophan metabolism have been related to several diseases, including neurodegeneration, gastrointestinal disorders and cancer. Tryptophan metabolism underlies tissue specific regulation [20], resulting in a remarkable difference in metabolite concentrations and fluxes. In our earlier analyses we focused on differential tryptophan pathway activity in two human tissues (brain and liver), as well as the metabolite exchange between these tissues and its consequences for neurodegenerative diseases and potential treatments [19]. We implemented a data driven modelling approach [21, 22] by scaling maximal reaction velocities based on expression data [19]. By integrating data from a tissue specific expression profiling study [23], we showed that we were able to quantitatively reproduce metabolite concentrations measured in vivo as well as qualitative flux changes reported upon treatment with inhibitors specific for enzymes of sub-pathways in mice. Since the tryptophan catabolite kynurenine has been associated with increased malignancy in brain tumours [24], we recently applied our model to calculate changes in tryptophan metabolism in different subtypes of breast cancer patients using RNA-sequencing datasets from The Cancer Genome Atlas (TCGA: https://cancergenome.nih.gov). We were able to show that our predictions are in agreement with kynurenine concentrations measured in patients [25]. Thus, incorporating theoretical model predictions allows us to predict patient specific diagnostic markers important for further treatment, emphasising the need for easily accessible data integration tools.

### Tissue specific differences in tryptophan metabolites

**Fig. 2:**
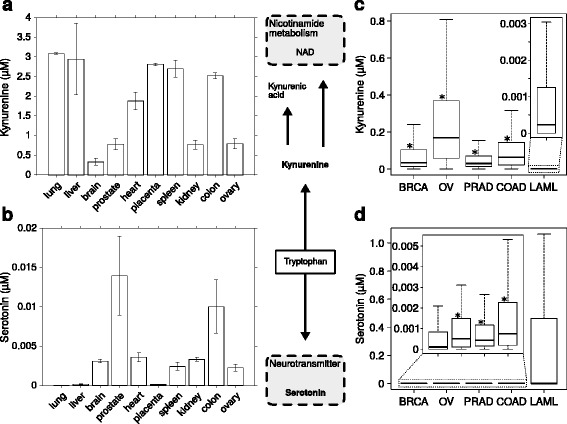
Calculation of steady state concentrations of kynurenine and serotonin. A simplified scheme of tryptophan metabolism (including network location of kynurenine and serotonin) is depicted in the middle. All depicted kynurenine and serotonin concentrations were calculated by integrating gene expression data into a model of mammalian tryptophan metabolism [19]. **a**, **b** Calculated steady state concentrations of kynurenine (**a**) and serotonin (**b**) for models of ten different tissues [23]. Bar height equals mean, error resembles standard error of the mean (SEM), three replicates per tissue. **c**, **d** Calculated steady state concentrations of kynurenine (**c**) and serotonin (**d**) for models derived by intergration of expression data from five different cancer types (data downloaded from the cancer genome atlas TCGA). *Asterisks* show statistically significant differences in comparison to acute myeloid leukemia. (BRCA: Breast invasive carcinoma, *n*=805; OV: Ovarian serous cystadenocarcinoma, *n*=228; PRAD: Prostate adenocarcinoma, *N*=441; COAD: Colon adenocarcinoma, *n*=421; LAML: Acute myeloid leukemia, *n*=51; Box plots represent median and the 75% and 25% percentiles. Whiskers extend to the most extreme data point which is no more than 1.5 times the interquartile range from the box. Outliers are omitted for the sake of visibility)

Kynurenine and serotonin are products of competing branches of tryptophan metabolism (see simplified pathway scheme Fig. 2). Their ratio has been recognized to be important in depressive disorders, especially in the context of chronic inflammation [26].

Here we extend our earlier analysis [19] to better understand the tissue specific activity of tryptophan metabolism. For this purpose we integrated a published tissue specific gene expression dataset from 32 human tissues [23] (dataset: https://www.ncbi.nlm.nih.gov/geo/query/acc.cgi?acc=GSE7905) and calculated steady state concentrations of kynurenine and serotonin with SBMLmod.

Our modelling approach predicts that liver as well as immuno-active tissues like lung and spleen have high kynurenine concentrations (Fig. 2 a). In lung and spleen the activity of the kynurenine pathway depends on the induction of indoleamine 2,3-dioxygenase (IDO), especially during infection (for review cf. [27, 28]). The tryptophan pathway activity in the liver is regulated via the expression of tryptohpan 2,3-dioxygenase (TDO) catalysing the same reaction as IDO. TDO is furthermore known to be down-regulated when peripheral kynurenine levels are increased, for example during infection [29]. Changes in tryptophan metabolism during pregnancy have been described previously, for example high expression of IDO in the placenta might play a role in immune tolerance [30]. The calculated concentrations for the placental model resemble these observations. In contrast, brain tissues are predicted to have a low activity of the kynurenine branch in healthy individuals. This is reasonable as several intermediates of the kynurenine branch are known to be neurotoxic [31].

Serotonin production is predicted to be high in neuroendocrine tissues such as the prostate, but low in tissues with high kynurenine pathway activity (Fig. 2 b) due to the competition for the substrate tryptophan. The comparatively high serotonin production in prostate epithelial cells has been described in the literature [32]. Our modelling approach furthermore predicts serotonin production to be high in the colon, but in this tissue the kynurenine route of the tryptohpan pathway is also partially active. This dual pathway activity in the colon has been reported earlier [33] and imbalances between the two branches might cause the development of irritable bowel syndrome [34, 35].

For a full overview of steady state concentrations of kynurenine and serotonin in all 32 available tissues see Additional file 4: Figure S4. Details on the statistical procedure are provided in the Additional file 5: S5. All pairwise statistical test results between all tissues are provided in Additional file 6: Table S6. The full dataset, mapping file and model are provided in Additional file 2: S2 and as example files in the web application (limited to the 10 tissues presented in Fig. 2 a and b).

### Different cancer types possess notable differences in kynurenine and serotonin concentrations

In a second analysis, we integrated RNA-sequencing data from approx. 2000 patients available at TCGA (https://cancergenome.nih.gov; corresponding TCGA-IDs are provided in Additional file 7: S7). Using this approach, we predicted activation of the kynurenine pathway and thus increased kynurenine production for ovarian, prostate and colorectal cancer (Fig. 2 c). Whereas the serotonin branch appears to be activated in acute myeloid leukemia, the kynurenine branch is largely inactive (Fig. 2 d). This is supported by statistical analysis showing that the distributions of kynurenine and serotonin concentrations are significantly different between the different cancer types (Kruskal-Wallis test, *p*=1.5e-93 and *p*=7.2e-33, respectively). Subsequent pairwise comparison reveals that kynurenine concentrations are predicted to be significantly higher in breast, ovarian, prostate and colorectal cancer as compared to acute myeloid leukemia (Fig. 2, Bonferroni corrected *p*-values 2.6e-42, 2.3e-83, 8.2e-32, 3.5e-56, respectively). In contrast, pairwise comparison of serotonin concentrations among different cancer types shows significantly lower concentrations of serotonin in ovarian, prostate and colorectal cancer, but not in breast cancer, when compared to acute myeloid leukemia (Fig. 2, Bonferroni corrected *p*-values 1.1e-4, 2.2e-5, 1.7e-9, 1, respectively). This is in agreement with known changes in these tumour types [24, 25, 36, 37]. An extended statistical analysis is provided in Additional file 8: Table S8.

## Conclusion

We presented SBMLmod, an SBML model modification and simulation tool. The platform-independent web application of SBMLmod allows for the automated integration of experimental data into theoretical models without requiring programming knowledge from the user. SBMLmod has two major advantages over existing methods: first, data integration and analysis are possible with a minimal number of user required operations; second, all operations can be performed without further software or programming dependencies. The easy accessibility of SBMLmod is accomplished by focusing on a limited number of essential model modification functions. These are complemented with steady state calculations of metabolite concentrations and fluxes. Additional flexibility is offered by accessing the application as a web service., which allows to further optimise and accelerate data integration and subsequent theoretical analyses.

Even though SBMLmod minimises the effort required by the user, we emphasise the need to ensure an accurate reaction or gene identifier mapping. Though models of sizes up to a genome-scale can be calibrated and simulated, ensuring correct mapping files is increasingly challenging if thousands of identifiers must be handled. Furthermore, increased simulation times due to the size of large models alone have to be considered; thus, SBMLmod is more suited for the manipulation and simulation of small and medium scale models. Of note, SBML is an XML format and is therefore not designed to be human readable. This can be compensated for by making use of the recently developed SBtab [38], which allows users to read and filter SBML files for relevant information such as metabolite names or reaction identifiers.

We demonstrated the usefulness of SBMLmod by calibrating a given tryptophan model to recapitulate an existing analysis of tryptophan metabolism and by evaluating the steady state concentrations of kynurenine and serotonin, two potential prognostic biomarkers in different diseases including cancer. We expect that SBMLmod will contribute to further improve data integration into modelling approaches especially with respect to accessibility.

## Availability and requirements:

**Project name:** SBMLmod

**Project home page:** http://sbmlmod.uit.no and https://github.com/MolecularBioinformatics/sbml-mod-ws

**OS:** any

**Programming language:** Python 2.7

**Licence:** GNU General Public License v2.0

## Additional files

Additional file 1 S1 — documentation. Documentation of the usage and file formats of SBMLmod. Also available at http://sbmlmod.uit.no. (PDF 49 kb)

Additional file 2 S2 — example files. Zipped example files usable to review specific data file format or to check SBMLmod web application and service functionality. These files resemble the first use case with 32 tissues in the manuscript. Note that mapping files, the SBML model and the data file limited to 10 tissues, can also be downloaded from the web application (http://sbmlmod.uit.no) using the download link at the lower part of the webpage under ‘Example Files’. (ZIP 32 kb)

Additional file 3 Figure S3 — visualisation of results by the web application. Example for the result visualisation of the 10 tissues (shown in Fig. 2 a and b) that is provided as part of the web application. (PDF 87 kb)

Additional file 4 Figure S4 — steady state concentrations of all 32 tissues. This figure provides a comprehensive overview over all 32 tissues that have been analysed. The figure complements Fig. 2 a and b, where 10 selected tissues are shown. (PDF 103 kb)

Additional file 5 S5 – details of statistical analysis. This file provides details of statistical analysis applied for the two use cases in this manuscript. (PDF 71 kb)

Additional file 6 Table S6 — detailed statistical results for dataset of 32 tissues. This file provides ANOVA and post hoc pairwise test statistics for all 32 tissues that have been analysed and described in the subsection ’Tissue specific differences in tryptophan metabolites’. (XLS 71 kb)

Additional file 7 S7 — TCGA sample IDs. List of TCGA sample IDs used to calculate the results presented in Fig. 2 c and d. (TXT 76 kb)

Additional file 8 Table S8 — statistics for TCGA dataset. This table provides ANOVA and post hoc pairwise test statistics for the TCGA data application as described in section ‘Different cancer types possess notable differences in kynurenine and serotonin concentrations’. (XLS 11 kb)

## Abbreviations

IDO: Indoleamine 2,3-dioxygenase
TDO: Tryptohpan 2,3-dioxygenase
