# Supplementary material for: SBMLmod: a Python-based web application and web service for efficient data integration and model simulation
Source: BMC Bioinformatics. 2017 Jun 24;18:314. doi: 10.1186/s12859-017-1722-9 (PMC5483284; doi:10.1186/s12859-017-1722-9)
Supplement: Supplementary file 1 — S1 — documentation. Documentation of the usage and file formats of SBMLmod. Also available at http://sbmlmod.uit.no. (PDF 49 kb) [file 12859_2017_1722_MOESM1_ESM.pdf]

Help

SBMLmod is a web application that allows the integration of various data into a given SBML model.
Accepted data cover common gene expression, proteomics or metabolomics data.
The following help documentation includes a step by step guide on how to use the application, as well as a detailed out explanation of the required and accepted file formats.

1. Step by step guide
2. File formats
3. Example files
4. Merge modes
5. Cite us

Step by step guide

Step 1 - A. Input files

Locate and choose the files containing your model of choice as well as the data, which shall be automatically integrated into the model on your hard drive. If necessary, you may upload a mapping file, which maps identifiers of the data file to species or reaction IDs of the given model. Note that a mapping file is obligatory, if the IDs from your SBML model and the identifiers of your data file differ, but refer to the same gene or reaction (e.g. TP53 and p53). Please refer to the section File formats below for further details on file formats and options.

Example files are provided to demonstrate the usage of the application. To use the example files, tick the check box labelled **Example files** in the **Other options** subpanel and proceed to Step 2. Details about the example files are described here.

You may choose to activate the **Batch mode** in the subpanel **Other options**. In this mode, the model will not only be modified depending on one given data column in the uploaded data file, but on each data column separately. This will result in as many model files as given data columns in the uploaded data file. All subsequent tasks will be performed on all models.

Step 2 - B. Choose task

Depending on the files you uploaded in step 1, different tasks are available. All tasks need a model in SBML format.
Depending on your available data, you can adjust global and local reaction parameters or set initial species concentrations.
After pressing the **run task** button, the calibrated model is available for download. Additionally, further tasks can be chosen to calibrate the model further or to analyse steady states.
If one provides a model and data file, one may e.g. firstly, set the initial species concentration and adjust reaction parameters and secondly, run a steady state analysis on the resulting model file(s).
Note that providing only an SBML model file is already sufficient to run a subsequent steady state analysis.

Adjust reaction parameters

In an SBML model, parameters are defined either globally or locally. Global parameters can be used by all reactions, while local parameters can only be used by the particular reactions it has been defined within. To adjust these parameters using a data file, follow these instructions:

1. Choose global and/or local parameters by ticking the respective check boxes.
2. If you selected **Global parameters** the identifiers of the data file (or mapping file, if provided) will be matched with parameter identifiers annotated in the global section in the given SBML file. The associated values from the data file get assigned, either by replacing or scaling the existing value (see below for further descriptions).
3. If you have selected **Local parameters** you have to provide information about which parameter you want to adjust as well. This has to be specified in the text field labelled **Parameter to change**.
   Example: If the parameter to change describes enzyme concentrations and is named E_T in each reaction, then

type “E_T” into the text field next to **Parameter to change**. SBMLmod will attempt to find exactly this parameter for each enzyme, and uses the corresponding value from the data file to set the new value for E_T per enzyme. Hence, only the parameters will be changed that match exactly the user entry in **Parameter to change**.

4. Both global and local parameters can be either *replaced* or *scaled*.
   Scaling the parameters means that the old parameter value will be multiplied by the respective value provided in the data file. This may be useful if the model contains e.g. organ specific activities for the described enzymes, while you upload a data file with ratios of gene expression data from other tissues e.g. brain and liver.
   Replace consequently means that the respective data value from the data file will be used to overwrite the old value.
5. Finally, a specific merge mode must be selected. Merge mode is used only, if two or more entries in the data file map to the same parameter in the model. If the data file contains gene expression data, there could be several genes that map to the same reaction, hence the value to represent the parameter must be chosen or calculated. The different merging possibilities are described here.
6. Each task is executed by clicking the **run task** button in the “Adjust reaction parameters” panel.

Set initial species concentrations

Initial species concentrations may be set according to a given data file, e.g. concerning growth medium conditions. If the entries in the datafile do not map directly to the entries in the model file, a mapping file is again required.

To run this task, click the **run task** button in the “Set initial species concentrations” panel.

Steady state analysis

The uploaded or modified model(s) can be send to a COPASI web service to run a steady state analysis. For a description of COPASI, please refer to the COPASI website.

To run the steady state analysis, click the **run task** button in the “Steady state analysis” panel.

Visualising analysis output

Next to the steady state analysis a visualisation of the respective results is generated. You may update the visualisation with this task.

Species and reactions to be displayed may be selected in the respected field in the "Adjust graphical output" panel. Of note this panel only appears if a steady state analysis has been performed. Multiple entries can be selected by pressing the Ctrl key (Command key on Mac) or the shift key for a range of entries while selecting. If no entry is selected, all entries will be shown. The graph is limited to show the first 100 entries (alphabetically ordered).

If batch mode is used, as many values as there are columns in the data file are generated for every species concentration and reaction flux defined in the respective model. Of note, as the first column in the data file contains the identifiers, choosable data sets start with column two (first data set in second column). Values that belong to different data columns can be grouped by defining the groups in the field “Groups for visualisation”. If the field is left empty, all data columns are grouped. If the model outputs shall be grouped evenly across all models (based on respective data columns order), enter this number into the field (e.g. “2” to put outputs of models associated to column 2 and 3 in group 1, column 4 and 5 in group 2, etc.). You may also enter a more complicated grouping pattern. Two groups are separated by a semicolon “;”. Within a group, columns are separated by a comma “,”. Ranges may be given using a hyphen “-”. To define two groups consisting of the data columns 2, 3, and 4; and 5, 6, and 7, respectively, enter “2,3,4;5-7”, or simply “2-4;5-7”. Patterns can vary arbitrarily, e.g. “2,4,6;3,5,7” to define again two groups of model outputs associated to the respective data column numbers. To increase the usefulness and presentability of the visual output, calculated flux/metabolite values associated to a group are not shown, if all values are equal (model modifications had no influence on steady state calculation of the respective flux or metabolite value), unless all groups consist of identical values. In this case all values are plotted.

To adjust the graphical output, click the **run task** button in the “Adjust graphical output” panel.

File formats

Model (SBML) file

The model must be provided in Systems Biology Markup Languange (SBML) format. SBML is an xml derivate and designed to describe biological models of all scales. For further information, please check:

Michael Hucka, Frank T. Bergmann, Stefan Hoops, Sarah M. Keating, Sven Sahle, James C. Schaff, Lucian P. Smith and Darren J. Wilkinson: The Systems Biology Markup Language (SBML): Language Specification for Level 3 Version 1 Core. Available from Nature Precedings (2010) DOI: 10.1038/npre.2010.4959.1

Data file

The data file has to be a tab delimited text file, including one descriptive header row. The new model file(s) will be named according to the name given in the respective column of the header row. If batch mode is not used, state the column number to use for modifying the model. When using batch mode, each column will be used and multiple models will be produced, one per data column.
The columns should contain the following information:

Column 1:
: Data identifiers, e.g. gene identifiers in case of gene expression data, or metabolic compound identifiers in case of initial concentration data.

Column 2 - n:
: Data values, e.g. one column for each sample in the case of gene expression data.

The example files can be reviewed for the file format specifics as well.

Mapping file

The mapping file has to be a tab delimited text file with two or three columns. Each row describes a mapping of SBML model identifier and data identifier. If several data identifiers map to the same reaction identifier, then these can be listed in separate rows. The different entries will be merged according to the Merge Mode selected. **The mapping is case-sensitive!**
The columns should contain the following information:

Column 1:
: SBML model identifiers, e.g. global parameter identifiers, reaction IDs (for integration of local parameters), or species identifiers, depending on which method is used for the integration. Note that you can also add identifiers of several model aspects in one mapping file to change both global and local parameters in one run.

Column 2:
: Data identifiers. These identifiers must match exactly the identifiers in Column 1 of the data file.

Column 3 (optional):
: Key words COMPLEX and/or ISO as annotation for proteins that are either isoforms or part of a complex. This column is only needed if “Custom” is selected as Merge Mode.

Example files

Four files are provided as example files.

1. **sbml_model.xml**: A valid SBML model.
2. **mapping.txt**: A mapping file of reaction identifiers.
3. **reaction_data.csv**: Data used for replacing (or scaling) reaction associated parameters in a global or local manner. Since the identifiers do not match the identifiers in the SBML model, the mapping file is used.
4. **species_data.csv**: Data used for resetting the initial concentrations of certain species in the SBML model. The identifiers in the data file already match the identifiers in the SBML model, hence no mapping file is required.

Note: To demonstrate warning feedback we used only one mapping file to match up identifier names between the model

and the data files. Warning messages will be displayed, when testing either the global or the local **adjust reaction parameter** option. These warnings describe not matchable reaction identifiers.
This is due to the fact that global and local identifiers may not have the same naming, that is, a mapping name for a global parameter may not be found when performing a local parameter adjustment and vice versa.
We therefore recommend to use one specific mapping file for each parameter adjustment operation, though mapping information can be combined in one file as demonstrated.

Merge modes

Merge modes are only considered, when two or more entries in the data file match to the same parameter.

Max
: The highest data entry is chosen to scale or replace the parameter value.

Min
: The lowest data entry is chosen to scale or replace the parameter value.

Sum
: The sum of the data entries is chosen to scale or replace the parameter value.

Mean
: The mean of the data entries is chosen to scale or replace the parameter value.

Median
: The median of the data entries is chosen to scale or replace the parameter value.

Custom
: Chooses either Min or Sum according to third column entry in mapping file:
COMPLEX - min
ISO - sum
Reasoning:
Iso-enzymes may be expressed in different tissues, yet are often represented by one reaction in SBML models. During the integration this can be resolved by summing up the gene expression values for iso-enzymes that map to the same reaction. For enzyme complexes the amount of active enzyme may be related to the availablility of only specific, e.g. regulatory, subunits. The 'Custom' option therefore uses the gene with the lowest expression value to represent the activity of the whole enzyme complex. To use this method a third column must be present in the mapping file containing either COMPLEX and/or ISO annotations.

How to cite

Please cite our URL sbmlmod.uit.no, when using SBMLmod in your analysis.
