# Supplementary material for: SBMLmod: a Python-based web application and web service for efficient data integration and model simulation
Source: BMC Bioinformatics. 2017 Jun 24;18:314. doi: 10.1186/s12859-017-1722-9 (PMC5483284; doi:10.1186/s12859-017-1722-9)
Supplement: Supplementary file 3 — Figure S3 — visualisation of results by the web application. Example for the result visualisation of the 10 tissues (shown in Fig. 2 a and b) that is provided as part of the web application. (PDF 87 kb) [file 12859_2017_1722_MOESM3_ESM.pdf]

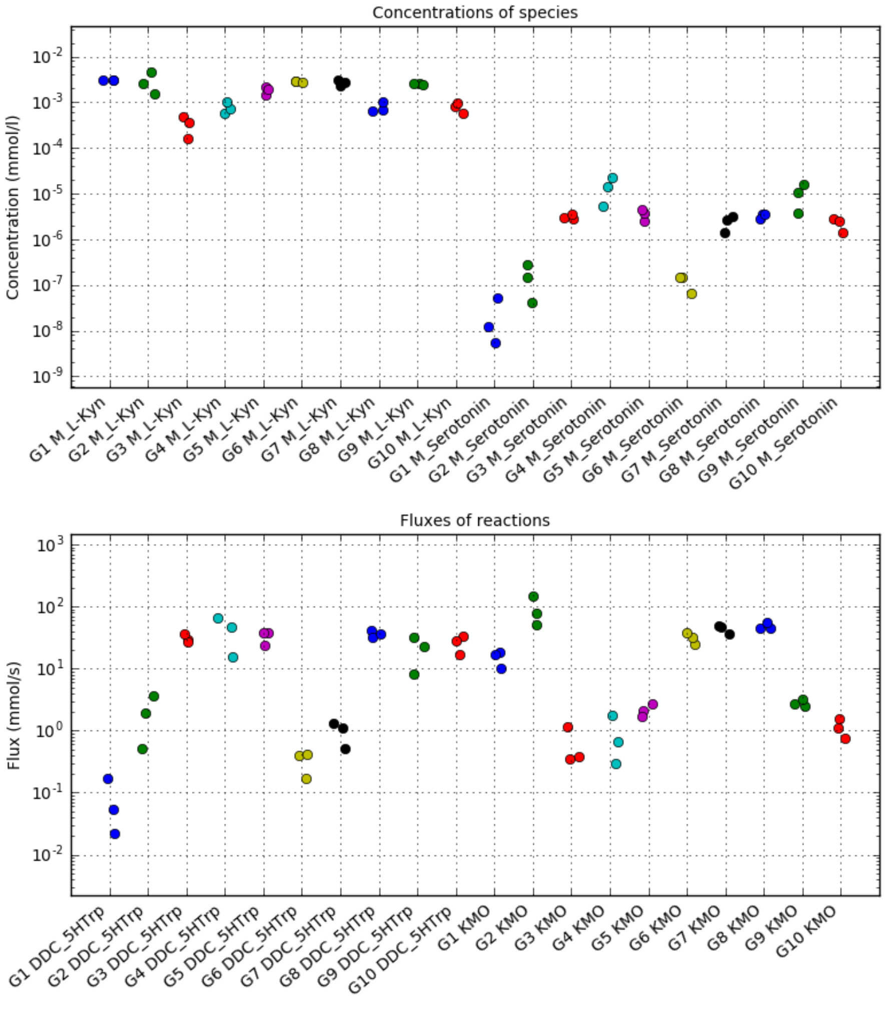

Figure S3: Example for result visualisation provided by the web application. Non-zero species concentrations and reaction fluxes are visualised by dots, which are grouped based on the definition provided by the user. G1 through G10 prefix denote lung, liver, brain, prostate, heart, placenta, spleen, kidney, colon and ovary tissue, respectively. Metabolites and fluxes are ordered alphabetically. (Abbreviations: M_ is a the prefix set for metabolites in the model used. DDC_5HTRP: aromatic-l-amino acid decarboxylase using 5-hydroxytryptophan as substrate; KMO: kynurenine 3-monooxygenase)
