# Supplementary figures and images for: SBMLmod: a Python-based web application and web service for efficient data integration and model simulation
Source: BMC Bioinformatics. 2017 Jun 24;18:314. doi: 10.1186/s12859-017-1722-9 (PMC5483284; doi:10.1186/s12859-017-1722-9)

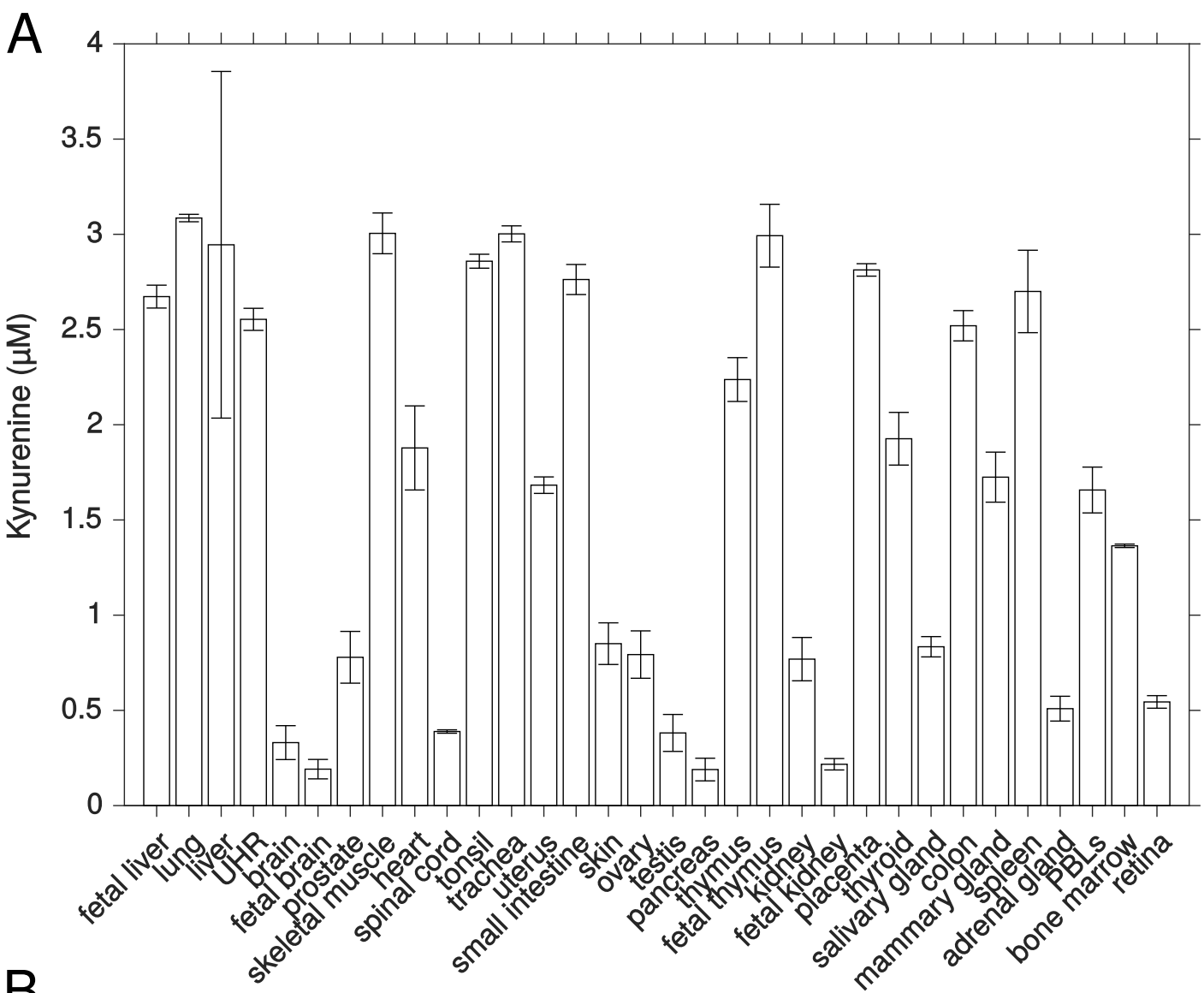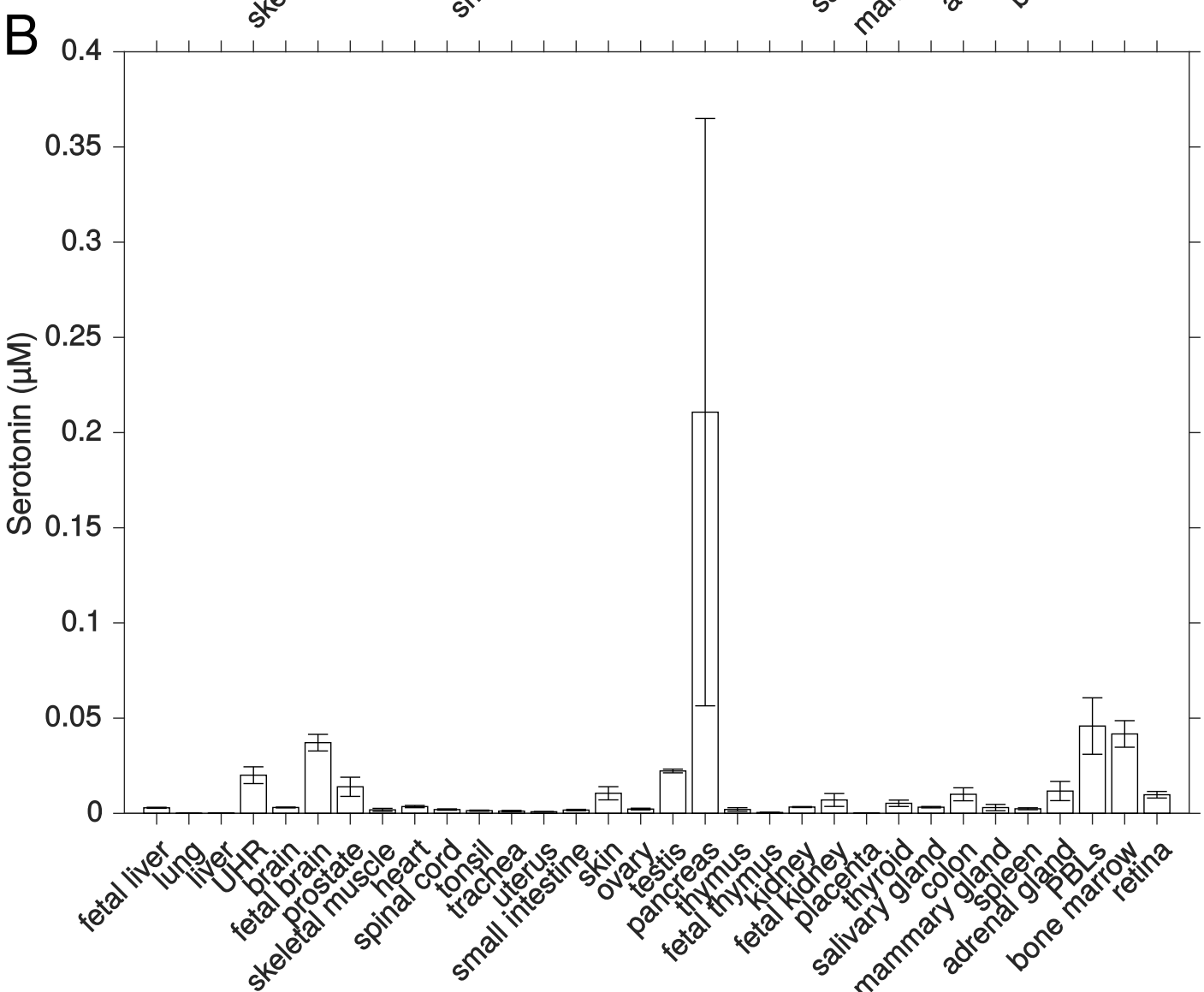

Supplement: Supplementary file 4 — Figure S4 — steady state concentrations of all 32 tissues. This figure provides a comprehensive overview over all 32 tissues that have been analysed. The figure complements Fig. 2 a and b, where 10 selected tissues are shown. (PDF 103 kb) [file 12859_2017_1722_MOESM4_ESM.pdf]
