# Supplementary material for: SBMLmod: a Python-based web application and web service for efficient data integration and model simulation
Source: BMC Bioinformatics. 2017 Jun 24;18:314. doi: 10.1186/s12859-017-1722-9 (PMC5483284; doi:10.1186/s12859-017-1722-9)
Supplement: Supplementary file 5 — S5 – details of statistical analysis. This file provides details of statistical analysis applied for the two use cases in this manuscript. (PDF 71 kb) [file 12859_2017_1722_MOESM5_ESM.pdf]

Additional File S5 – statistical procedure

Statistical analysis has been conducted with MATLAB version R2015b. Next, details and test specific MATLAB functions (set in `'typewriter'`) are outlined.

Use case 1

The tissue dataset has been downloaded from https://www.ncbi.nlm.nih.gov/geo/query/acc.cgi?acc=GSE7905 as indicated in the main text. One-way ANOVA (`'anova1'`) had been applied to check for differences of variances of kynurenine and serotonin concentrations among different tissues. Subsequent multiple comparison test with applied Bonferroni multiple test correction (`'multcompare'`) was applied to check for pairwise statistical significance of differences in free concentrations of kynurenine or serotonin among different tissues. Results are detailed out in Additional File: Table S6.

Use case 2

RNASeq data were downloaded from http://cancergenome.nih.gov. The corresponding patient identifiers are listed in Additional File S7. For each patient a separate model was generated and respective steady state metabolite concentrations calculated. The initial tryptophan concentration was set to 5 µM, which corresponds to the median concentration of free tryptophan measured in blood [1].

As Komolgorov-Smirnov test (`'kstest'`) and Levene test (`'vartestn'` with 'TestType' set to 'LeveneAbsolute' option) revealed that assumptions for one-way ANOVA are violated, non-parametric Kruskal-Wallis test has been applied to check for differences of variances of kynurenine and serotonin concentrations between different cancer types. Finally, a multiple comparison test with Bonferroni multiple test correction (`'multcompare'`) was applied to check for pairwise statistical significance of differences in free concentrations of kynurenine or serotonin between different cancer types.

References

[1] Chen, Y., Guillemin, G.J.: Kynurenine pathway metabolites in humans: disease and healthy States. Int J Tryptophan Res **2**, 1–19 (2009)
